# Why Osteosarcopenia Matters: Clinical and Investigative Implications

**DOI:** 10.1111/eci.70221

**Published:** 2026-05-14

**Authors:** Erik Luiz Bonamigo, Marcella Nora Maia, Renata Pinheiro Cavallaro de Oliveira, Régis Gemerasca Mestriner, Ivan Aprahamian, Gustavo Duque

**Affiliations:** ^1^ Bone, Muscle & Geroscience Group Research Institute of the McGill University Health Centre Montreal Quebec Canada; ^2^ Hospital São Lucas da Pontifícia Universidade Católica Do Rio Grande Do Sul Porto Alegre Rio Grande do Sul Brazil; ^3^ Departamento de Medicina Interna, Divisão de Geriatria Faculdade de Medicina de Jundiaí Jundiaí São Paulo Brazil; ^4^ Instituto de Geriatria e Gerontologia (IGG‐PUCRS) Pontifícia Universidade Católica Do Rio Grande Do Sul Porto Alegre Rio Grande do Sul Brazil; ^5^ Division of Geriatric Medicine, Department of Medicine McGill University Montreal Quebec Canada

**Keywords:** aging, bone–muscle crosstalk, frailty, osteoporosis, osteosarcopenia, sarcopenia

## Abstract

**Background:**

Osteosarcopenia, the combination of osteoporosis and sarcopenia, is a geriatric syndrome linked to functional decline, falls, and fragility fractures. The interaction among bone, muscle, and their shared pathophysiology is driven by mechanical, metabolic, and hormonal factors. With global population aging and increasing healthcare demands, early detection of osteosarcopenia has become essential.

**Methods:**

This narrative review summarizes current evidence on the epidemiology, pathophysiology, clinical findings, diagnosis, and treatment of osteosarcopenia, based on international consensus guidelines, large‐scale population cohorts, interventional studies, and translational research.

**Results:**

The occurrence of osteosarcopenia reflects the combined impact of low bone mineral density (BMD), loss of muscle mass and strength, and age‐related metabolic changes such as chronic inflammation, lipotoxicity, and disruptions in tryptophan (TRP) metabolism. Diagnostic evaluation requires combining bone assessment through Dual‐energy X‐ray Absorptiometry with functional and structural evaluations of sarcopenia, using criteria established by the European and global consensus on Sarcopenia. Management includes established pharmacological therapies for osteoporosis, while for sarcopenia, to date, it is mainly based on resistance exercise and adequate protein intake. Supplementation with protein, leucine, vitamin D, calcium, and creatine may further enhance outcomes. Promising emerging strategies include hormonal modulators, anti‐inflammatory agents, metabolic pathway–oriented therapies, and cell‐based interventions.

**Conclusions:**

Osteosarcopenia significantly raises the risk of falls, fractures, disability, and death. Effective management requires a comprehensive treatment approach that targets both bone and muscle decline. Further research is necessary to refine diagnostic criteria and assess the success of combined interventions through clinical trials.

## Graphical abstract

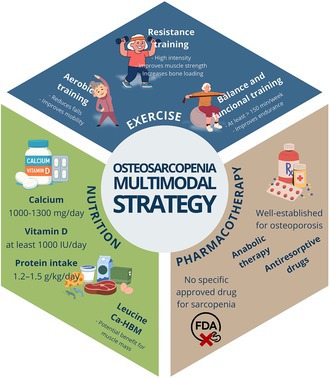

## 1 Introduction

Osteosarcopenia is a combination of osteoporosis and sarcopenia, and it is biologically plausible given the close mechanical, biochemical, and metabolic communication between bone and muscle [1]. Its pathophysiology is evolving, complex, and still under research. It involves the integrated interaction of bone, muscle, and adipose tissues, which collectively maintain musculoskeletal homeostasis [2].

Epidemiological data confirm that fractures in older adults pose a significant global public health challenge; therefore, early diagnosis is crucial for identifying at‐risk patients promptly. This geriatric syndrome is linked to falls and fragility fractures [3] and the presence of osteosarcopenia increases the likelihood of adverse outcomes [4, 5] hospitalizations [6] nursing home admissions [7] and mortality, as well as higher healthcare costs [8] leading to a decrease in healthspan. Since individual risk of fragility fracture can be predicted and these fractures may be prevented through effective interventions, this review aims to summarize concepts, pathophysiology, diagnosis, and treatment approaches for osteosarcopenia, offering a concise and practical medical update.

## 2 Definition and Epidemiology

Osteoporosis is a complex disease that has gained increasing attention over recent decades due to its clinical relevance and significant consequences in a rapidly aging world [9]. In the 1990s, one of the first official definitions was established by a multidisciplinary expert panel, which described it as a “systemic skeletal disease characterized by low bone mass and microarchitectural deterioration of bone tissue, with a consequent increase in bone fragility and susceptibility to fracture” [10]. The following year, the World Health Organization (WHO) Study Group, sponsored by the European Foundation for Osteoporosis and Bone Disease [11]. refined the definition and included the bone mineral density (BMD) as a diagnostic criterion, thereby allowing to start treating even before the occurrence of fractures [12].

Epidemiological data confirm that fractures in older adults represent a major global public health challenge. In 2019, according to the Global Burden of Disease (GBD) study, which provides a comprehensive assessment of the incidence, prevalence, mortality, and disability for various diseases, injuries, and risk factors in 204 countries, there were 178 million new fractures worldwide and 455 million prevalent cases of acute or chronic fracture‐related symptoms. The data covers age ranges from 0 to 6 days to 95 years or more [13]. In comparison, 1990 data estimated 338,000 hip fractures in men and 917,000 in women, totaling 1.26 million, based on a projection of demographic data from the World Health Organization divided into age ranges from 5 years to 80 years, and above that categorized as 80 years or older [14]. In 2019, fractures accounted for 25.8 million years lived with disability (YLDs) a 65.3% increase in absolute YLDs since 1990 [13].

The etymology of sarcopenia means “loss of muscle,” but, of course, this literal meaning is not enough to describe a complex condition. Although “sarcopenia” was recognized as the “decline in strength and function,” which increases the “risk of disability, falls, and mortality,” it was only in 2019 that the European Working Group on Sarcopenia in Older People (EWGSOP2) established “low muscle strength” as a primary criterion for diagnosing probable sarcopenia. Regarding muscle mass, the EWGSOP2 proposed using Dual‐energy X‐ray Absorptiometry (DXA) to measure lean mass as a surrogate for muscle mass. It has the limitation that it measures technically fat‐free mass, excluding bone, not solely muscle. While reaching consensus on the definition of sarcopenia has been difficult, the Global Leadership Initiative in Sarcopenia (GLIS) has provided clear guidance and a conceptual definition by harmonizing regional definitions following a global Delphi consensus process, which proposed measuring muscle mass, strength and muscle‐specific strength (strength/mass) as being highly predictive of adverse outcomes [6]. This strategy will make it easier to identify sarcopenia in clinical practice and will support the development of new treatments for this condition.

When osteoporosis/osteopenia and sarcopenia coexist, this condition is often called osteosarcopenia [15]. This overlap makes biological sense given the close mechanical, biochemical, and metabolic interactions between bone and muscle [1] where a decline in one tissue induces deterioration in the other. Indeed, patients with sarcopenia are 18 times more likely to have osteopenia or osteoporosis compared to age‐matched controls, as shown in multiethnic groups comprising 17,891 participants [16]. A Swedish population‐based study, for instance, involving over 3000 older adults, found that individuals with probable or confirmed sarcopenia had lower BMD and compromised bone structure at various sites [17]. Additionally, age‐specific fracture rates in 2019 were highest among the oldest‐old, with those aged 95 and above experiencing over 15% of new fractures [13]. Due to the increasing and aging global population, the prevalence of osteosarcopenia is expected to grow [18] which could lead to more falls, fractures, disability, and hospitalizations worldwide [8].

## 3 Pathophysiology

**FIGURE 1:**
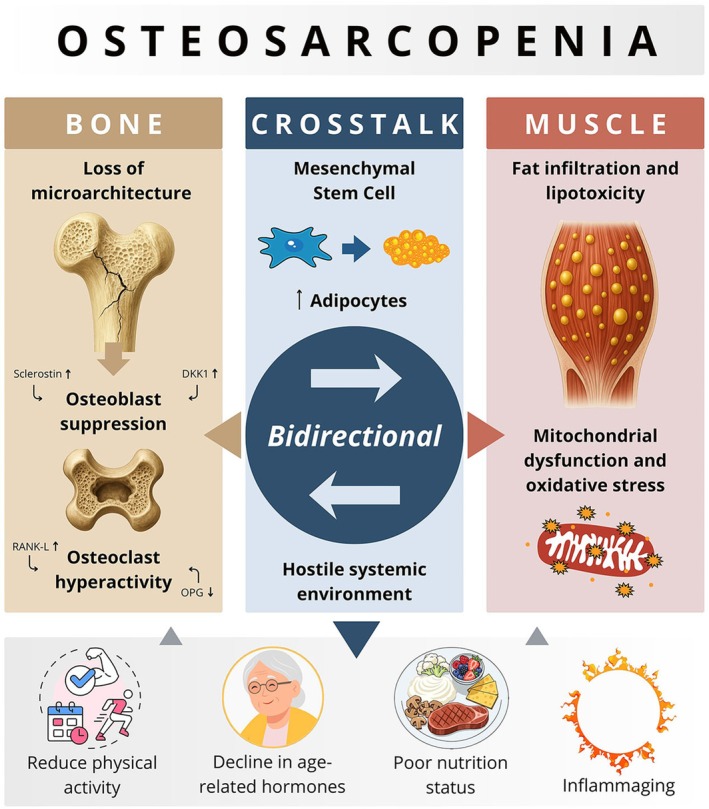
Pathophysiology of osteosarcopenia. DKK1, dickkopf‐related‐protein‐1; OPG, osteoprotegerin; RANK‐L, receptor activator of nuclear factor κ‐B ligand.

The pathophysiology of osteosarcopenia (Figure 1) is emerging, complex, and still under investigation. It involves integrated interaction of bone, muscle, and adipose tissues, which together maintain musculoskeletal homeostasis [2]. Sarcopenia's aetiology is multifactorial and includes age‐related changes in the immune and endocrine systems (with hormonal imbalances, chronic low‐grade inflammation—inflammaging—and increased oxidative stress). Another significant key is the disturbance of protein turnover, when catabolism exceeds synthesis and increases adiposity, particularly intra‐ and inter‐muscular fat deposition [19].

Osteopenia and osteoporosis' physiopathology is characterized by an age‐related decline in BMD and deterioration of its microarchitecture. These changes may be attributed to an imbalance between a reduced number of bone‐forming cells (osteoblasts) and increased activity of bone‐resorbing cells (osteoclasts), resulting in a net loss of bone mass. Reduced physical activity and poor nutritional status (insufficient intake of protein, vitamin D and calcium) further contribute to the aetiology and development of osteosarcopenia [19]. A general overview should begin with a comprehensive understanding of the bone's function, cellular components and their interactions with other systems.

The bone's matrix (containing organic compounds) serves as the environment in which external factors such as hormones, proteins, vitamins, and growth factors interact with specialized cells (osteoclasts, osteoblasts, and osteocytes) in a well‐coordinated manner to regulate bone mass. Bone mass depends on continuous remodelling, which is coordinated by factors secreted by osteocytes and osteoblasts that control osteoclastic activity and bone resorption. This process, which relies on the good cooperation of these cells, results in peak bone mass between 25 and 30 years of age. After that, changes in cellular distribution with age cause a gradual decline in bone mass at a normal rate of 0.5% per year. The main factors regulating these interactions are receptor activator of nuclear factor κ‐B ligand (RANKL) and osteoprotegerin (OPG). The former promotes osteoclast differentiation and activation, while OPG, produced mainly by osteoblasts, acts as a receptor for RANKL, inhibiting its action and, in turn, decreasing osteoclast activity. Osteocytes also influence bone formation by secreting sclerostin and dickkopf‐related‐protein‐1 (DKK1), both of which inhibit osteoblasts. Changes in these regulatory pathways contribute to osteoporosis.

Another key factor in osteosarcopenia syndrome is the population of mesenchymal stem cells (MSCs), which reside in connective tissues such as muscle, bone, and fat [8]. These cells can differentiate into adipocytes, myocytes, chondrocytes, and osteoblasts. When the bone marrow is young, MSCs mainly differentiate into osteoblasts rather than adipocytes; however, as age increases, this balance gradually shifts. An accumulation of fat in intramuscular tissue and bone marrow, independent of body mass index [2] is linked with aging and is a well‐recognized hallmark of this disease [8, 9, 10, 11, 12, 13, 14, 15, 16, 17, 18, 19, 20, 21]. It is also associated with the release of adipokines, which induce apoptosis of myocytes and osteocytes [21] lower osteoblast differentiation (reducing their function and survival), and stimulate osteoclastic activity [2].

Previous experimental studies have shown that marrow adipocytes produce substantial amounts of palmitic acid, which is toxic to bone‐forming osteoblasts in vitro [21]. The increased expression of this fatty acid in aged muscle and bone contributes to creating a lipotoxic environment in the surrounding tissue [8]. that ultimately contributes to the disrupted bone–muscle homeostasis. Additionally, recent studies have shown growing evidence that age‐related alterations in the “kynurenine pathway”, an essential route for tryptophan (TRP) catabolism, play a role in the pathogenesis of sarcopenia, osteoporosis, osteosarcopenia, and frailty [22, 23]. TRP is a dietary essential and an important amino acid involved in energy metabolism and DNA synthesis [23]. It is metabolized in multiple organs (including the gut) [22] through a series of intermediate steps, ultimately being converted into kynurenine (KYN) and subsequently into several end metabolites, including picolinic acid (PIC) – an anabolic compound with beneficial effects on muscle and bone – and quinolinic acid (QUIN) [22, 23].

This pathway may become dysregulated in older adults owing to chronic inflammation (inflammaging) and/or changes in gut microbiota, which affect the ability to produce anabolic metabolites such as PIC and QUIN. As a result, aging is associated with lower serum levels of these metabolites and a rise in potentially toxic metabolites that promote oxidative stress and cellular senescence in bone marrow stroma cells, impairing their differentiation into osteoblasts [22]. In summary, TRP‐metabolites like 3‐hydroxykynurenine (3‐HK), kynurenic acid (KYNA), and anthranilic acid (AA) negatively affect bone health. Conversely, high serum levels of 3‐hydroxyanthranilic acid (3‐HAA), PIC, QUIN, and nicotinamide adenine dinucleotide (NAD+) are linked to increased BMD and a reduced risk of fractures [22].

Mechanical stimuli are also important factors involved in the pathophysiology of osteosarcopenia. For instance, BMD is maintained through gravitational loading, which, when stimulated, leads to muscle hypertrophy and osteogenesis. Conversely, physical inactivity decreases both and simultaneously causes muscle and bone atrophy [8]. Importantly, in the context of sarcopenia, KYN induces muscle atrophy and lipid peroxidation, which negatively affect muscle mass and function [22]. Furthermore, muscle‐derived myokines, such as myostatin, also influence bone remodelling by promoting osteoclastogenesis and thereby increasing bone resorption. In addition, endocrine mechanisms also play a role in this disease: muscle and bone share similar metabolic processes that rely on vitamin D and amino acids. These nutrients influence protein turnover and collagen synthesis, supporting the formation of the bone matrix [8]. They also enhance calcium absorption and regulate cellular proteins and growth factors through insulin‐like growth factor 1 (IGF‐1), which inhibits parathyroid hormone secretion. The decline in age‐related hormones—including lower levels of testosterone, estrogen, IGF‐1, and growth hormone—accelerates muscle wasting and bone loss [8].

There are two additional mechanisms that may help in understanding this pathophysiology. The first, related to genetics, indicates that some gene polymorphisms are associated with muscle atrophy and bone loss, such as glycine‐N‐acyltransferase (GLYAT), methyltransferase‐like 21C (METTL21C), peroxisome proliferator‐activated receptor gamma coactivator 1‐alpha (PGC‐1α), and myocyte enhancer factor‐2 (MEF2C) [8]. Furthermore, mitochondrial dysfunction presents another potential mechanism that links various aspects of sarcopenia pathogenesis: high‐resolution microscopy and molecular analyses have revealed widespread alterations in mitochondrial biology, including reduced oxidative phosphorylation capacity, impaired dynamics, and compromised quality control systems. These changes decrease cellular energy production and influence muscle fibre type distribution and satellite cell function, promoting both oxidative stress and inflammatory responses. The interaction between mitochondrial dysfunction and other hallmarks of osteosarcopenia forms an interconnected system of cellular deterioration [24].

Finally, muscle–bone crosstalk constitutes an integral component of this pathophysiological process. Throughout life, the structure and function of bone and muscle are closely interconnected in a physiological process, reflecting a bidirectional integration of mechanical and biological signalling from embryonic development through aging. However, certain clinical conditions—such as aging, alterations in nutritional metabolism, physiological rhythms, chronic inflammation, physical inactivity, and systemic diseases—can disrupt this homeostasis, leading to modifications in muscle–bone communication and altering both the secretory profile of these tissues and their ability to respond to mechanical stimuli. As a result, anabolic signalling is impaired, and catabolic processes are favoured, contributing to the concomitant development of sarcopenia and osteoporosis, often manifested as osteosarcopenia [25]. Beyond generating mechanical stimuli for the skeletal structure, in the homeostasis of bidirectional integration, muscles also act as endocrine organs, secreting a variety of myokines that regulate bone, cartilage, and systemic metabolism. Key signalling pathways—including FGF, IGF1, IL‐6/7/15, irisin, myostatin, Tmem119, and osteoactive—coordinate osteogenesis, chondrogenesis, muscle growth, and energy homeostasis. Reciprocally, bone‐derived factors such as PGE2, sclerostin, FGF23, osteocalcin, and IGF1 exert endocrine and paracrine effects on skeletal, smooth, and cardiac muscle, influencing myogenesis, metabolism, and aging. Exosomes carrying non‐coding RNAs also mediate bidirectional muscle–bone communication, regulating cell survival, differentiation, inflammation, and regeneration [25].

## 4 Clinical Findings

**FIGURE 2:**
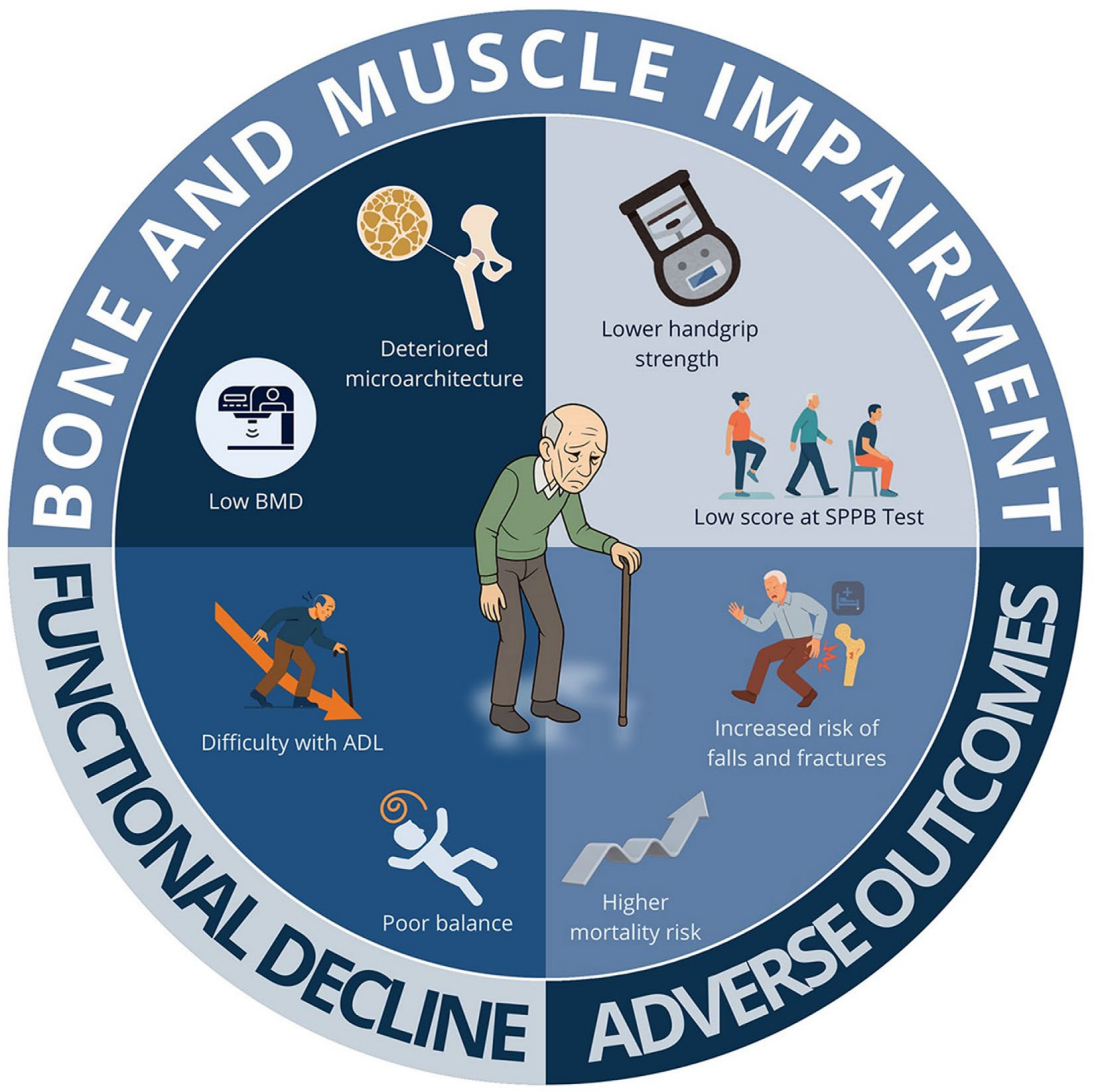
Osteosarcopenia: Clinical findings. BMD, bone mineral density; SPPB, short physical performance battery.

The clinical phenotype of osteosarcopenia is characterized by the coexistence of features from both bone and muscle impairment, specifically osteoporosis and sarcopenia (Figure 2). This term (first proposed in 2009 [15] and officially adopted in 2022 [20]) describes a syndrome marked by impaired physical performance (such as slow gait speed, poor balance, and prolonged Timed Up and Go test [TUG] times), low BMD (T‐score < −1.0 for osteopenia and ≤ −2.5 for osteoporosis), decreased muscle strength (like low handgrip strength), and reduced muscle or lean mass. The risk of functional disability is significantly increased in individuals with osteosarcopenia, particularly regarding Instrumental Activities of Daily Living (IADL) as measured by the Lawton Scale [26]. A previous study of 320 adults found that the osteosarcopenia phenotype was associated with a higher risk of functional disability than in individuals without osteopenia or sarcopenia, as assessed by the previously mentioned scale [26].

As is well known, by the sixth decade of life, there is a progressive decline in BMD estimated at 1%–1.5% per year. Simultaneously, muscle mass decreases by about 1% annually, while muscle strength declines at an even faster rate of 2.5%–3% per year. Furthermore, this combined bone‐muscle disorder affects bone microarchitecture and reduces bone strength [8]. Additionally, decreased muscle strength and reduced muscle mass are also characteristic features of this syndrome. These manifestations can include lower handgrip strength [27] decreased appendicular lean muscle mass, and impaired functional capacity, as evidenced by low scores on the Short Physical Performance Battery (SPPB Test).

Once demonstrated, arm‐cranking power is a risk factor for mortality, independent of muscle strength, physical activity, and muscle mass [28]. Patients with osteosarcopenia typically exhibit increased risk of falls and fractures [27, 28, 29, 30] especially hip and major osteoporotic fractures, as well as a higher likelihood of frailty and reduced life satisfaction [29]. As presented, among community‐dwelling males, those with osteosarcopenia are at greater risk of falls and fractures than individuals with preserved bone density and without sarcopenia [29]. Notably, the presence of osteosarcopenia increases the likelihood of adverse outcomes [4, 5] including frailty, hospitalizations [6] admissions to nursing homes [7] and mortality, as well as leading to a rise in healthcare expenditure [8].

## 5 Diagnosis

**FIGURE 3:**
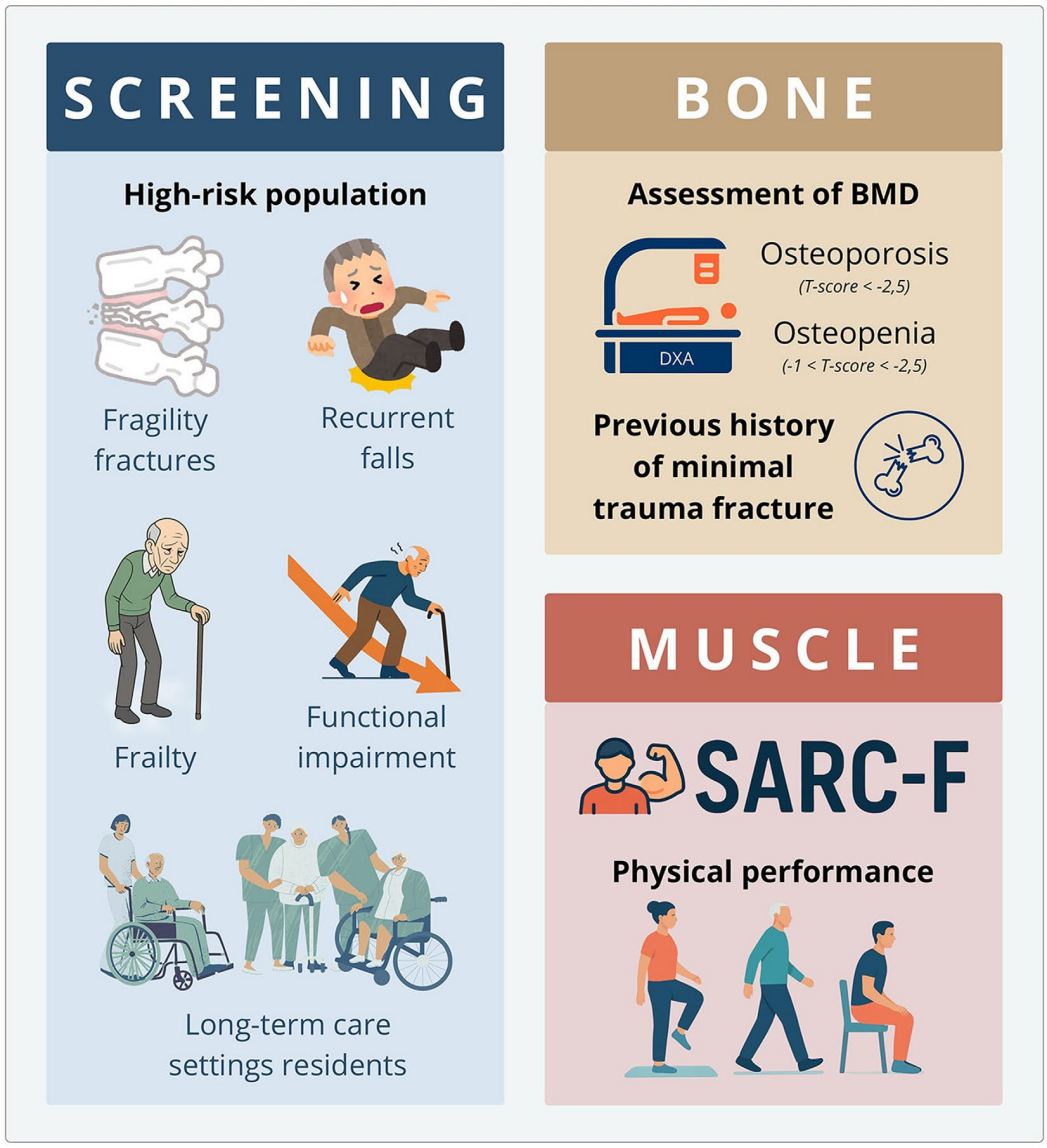
Osteosarcopenia: Diagnostic approach. DXA, dual‐energy X‐ray Absorptiometry; SARC‐F: A five‐item questionnaire evaluating the patient's perception of muscle strength, walking ability, rising from a chair, stair climbing, and history of falls.

Given that osteosarcopenia is a combined “bone and muscle” disease, its diagnosis (Figure 3) should aim to identify these patients as comprehensively and early as possible. As recognized, this geriatric syndrome is associated with falls and fragility fractures [3] which is why early diagnosis leads to better outcomes. For osteoporosis, as an isolated condition, BMD assessment by dual‐energy X‐ray absorptiometry (DXA) allows easier determination of its presence compared to sarcopenia, since DXA provides an objective measurement. The results are expressed as standard deviations (SD) from the expected mean value in healthy young individuals, and osteoporosis is defined as a BMD value 2.5 SD or more below (T‐score ≤ −2.5) [31]. A previous history of a minimal trauma fracture is also considered diagnostic of osteoporosis. Some groups have suggested that osteopenic subjects those with a BMD between −1 and −2.5 SD should be included in the criteria for osteosarcopenia, since 50% of minimal trauma fractures occur in this population, with their fracture risk most likely increased due to the presence of sarcopenia [8].

Regarding sarcopenia, as previously noted, diagnosing can be challenging, partly due to the lack of consensus on terminology. However, studies in various races and populations have shown that, regardless of the current definition used to diagnose sarcopenia, those with osteosarcopenia are at a higher risk for adverse outcomes [8]. The EWGSOP2 framework remains the most widely adopted worldwide for diagnosing sarcopenia [32]. The recommended approach begins with screening using the SARC‐F, a five‐item questionnaire that assesses the patient's perception of muscle strength, walking ability, rising from a chair, stair climbing, and falls history [33]. Next, muscle strength should be evaluated through handgrip dynamometry or the chair stand test. Physical performance can then be assessed via gait speed, the SPPB, or the TUG. Lean mass quantification is usually performed using DXA, based on total body lean tissue or appendicular skeletal muscle mass, or, when available, muscle mass can be measured through Magnetic Resonance Imaging and computed tomography; both considered standards but less often used due to limited availability and higher costs. Alternative methods include bioelectrical impedance analysis (BIA) or calf circumference measurement, though the latter is not generally recommended [32].

In addition to opportunistic case‐finding, there is a growing consensus that systematic screening for osteosarcopenia should be considered in high‐risk populations. These include older adults with prior fragility fractures, recurrent falls, frailty, functional impairment, or those residing in long‐term care settings. In such populations, the coexistence of low bone mineral density and impaired muscle function is frequent and often underdiagnosed, contributing to preventable adverse outcomes. Integrating muscle strength and physical performance assessments into routine osteoporosis and fracture risk evaluations may facilitate earlier identification and more comprehensive management of osteosarcopenia [8, 9, 10, 11, 12, 13, 14, 15, 16, 17, 18, 19, 20]. When present, a comprehensive approach is necessary—including investigating secondary causes and fall risk, optimizing comorbidities, and employing combined pharmacological and non‐pharmacological strategies. Furthermore, beyond the initial diagnosis, ongoing follow‐up is crucial for monitoring disease progression and adjusting the chosen therapy [8]. Thus, incorporating simultaneous assessments of bone density, muscle strength, and physical performance into routine geriatric and fracture prevention care may improve risk stratification and therapeutic decision‐making. Such an integrated approach aligns with the multidimensional nature of osteosarcopenia and supports personalized interventions aimed at preserving mobility, independence and healthspan in aging populations [34, 35]. In conclusion, clinicians should maintain a high suspicion for osteosarcopenia when evaluating patients with osteoporosis or sarcopenia.

Finally, it is important to note that the variety of consensus guidelines on the definition of sarcopenia and the tools used to diagnose the condition results in a heterogeneous prevalence, making it more challenging to identify individuals and provide appropriate treatment [36]. A cutoff point that underestimates prevalence leads to underdiagnosis and undertreatment, whereas overestimation increases the risk of excessive therapeutic intervention [37]. This emphasizes that the choice of measurement tools and threshold points is crucial [38].

## 6 Treatment

**FIGURE 4:**
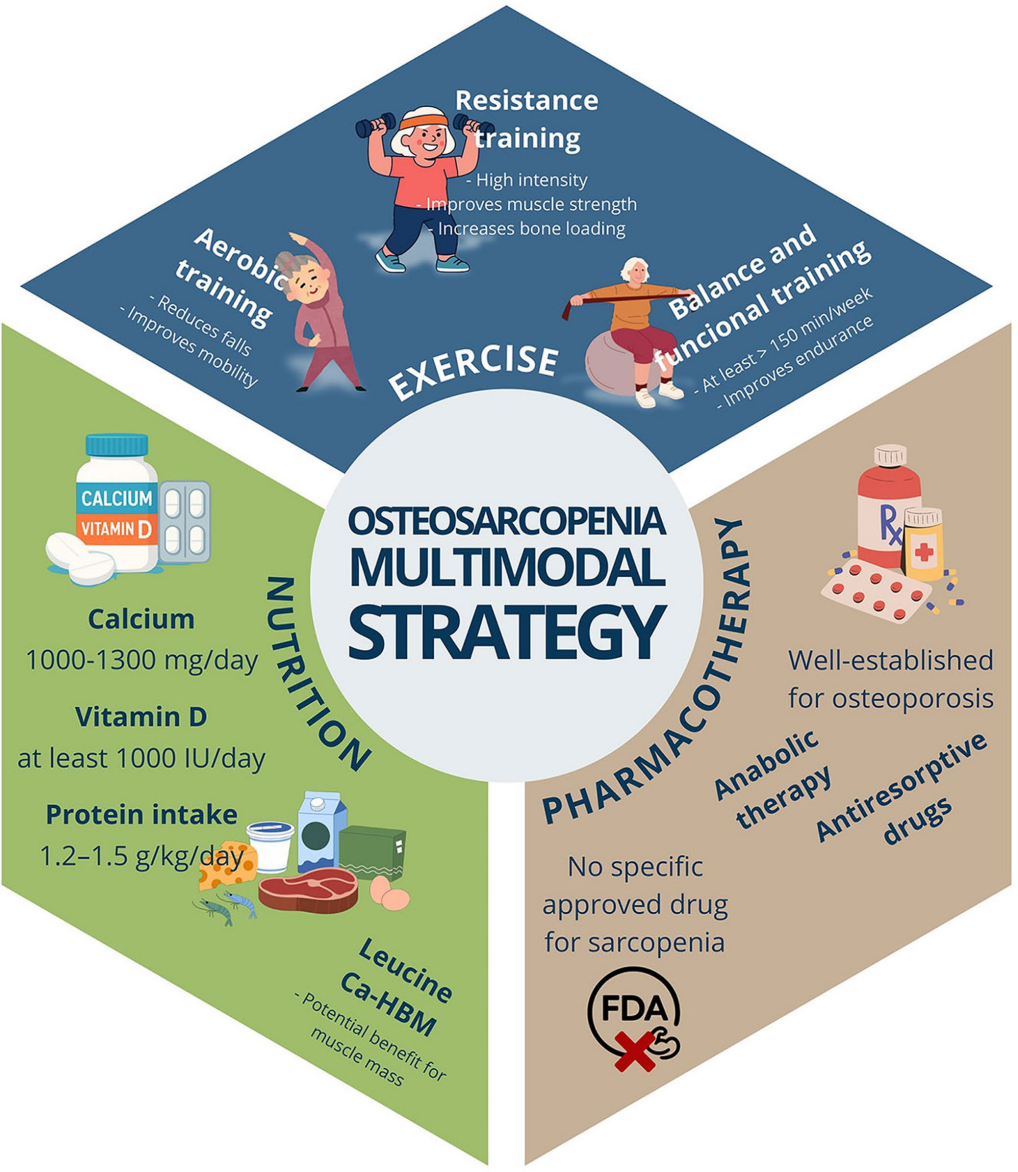
Osteosarcopenia: Non‐pharmacological and pharmacological treatment strategies.

There is a well‐established availability of pharmacological therapies for osteoporosis (Figure 4), including anabolic and antiresorptive agents. According to the National Osteoporosis Foundation, the use of antiresorptive or anabolic drugs is indicated when: adults have a minimal‐trauma hip or vertebral fracture; a DXA‐T score of ≤ −2.5 SD; or a FRAX 10‐year fracture risk of ≥ 3% at the hip or ≥ 20% for any other osteoporotic fracture. Before starting therapy, serum vitamin D levels should ideally be above 30 nmol/L [8]. In contrast, only non‐pharmacological interventions have been approved by the Food and Drug Administration (FDA) for sarcopenia, likely due to the relatively recent recognition of this clinical condition [24]. Consequently, osteosarcopenia is not currently recognized as a distinct pharmacological indication, and no approved therapies specifically target both bone and muscle deterioration simultaneously. Pharmacological treatment in this context remains guided by established osteoporosis criteria, while sarcopenia management relies predominantly on non‐pharmacological strategies. Ongoing research into agents targeting shared molecular pathways of bone and muscle aging may eventually reshape this paradigm, but such approaches remain investigational [39, 40].

Although antiresorptive therapies such as denosumab have been hypothesized to exert indirect effects on muscle through bone–muscle biochemical crosstalk, current clinical evidence remains insufficient to support consistent or clinically meaningful improvements in muscle mass, strength, or physical performance. Therefore, pharmacological treatment in osteosarcopenia should continue to be guided by established osteoporosis indications, while sarcopenia management relies primarily on exercise and nutritional interventions [41]. The first‐line recommendation for sarcopenia is non‐pharmacological approaches with multidomain lifestyle interventions. Both the Canadian Society for Exercise Physiology and the American College of Sports Medicine have similar exercise guidelines for older adults, recommending at least 150 min per week of moderate‐to‐vigorous aerobic activity combined with resistance training at least twice per week, including 8–10 exercises of 8–12 repetitions. Resistance exercise plus aerobics and/or balance training has been shown to improve patients' physical functions; also, it is known that physical activity increases the secretion of kynurenines (an anabolic component) [8, 9, 10, 11, 12, 13, 14, 15, 16, 17, 18, 19, 20, 21, 22, 23, 24, 25, 26, 27, 28, 29, 30, 31, 32, 33, 34, 35, 36, 37, 38, 39, 40, 41, 42, 43].

Beyond physical exercise, it is important to maintain a balanced diet rich in proteins [44] to support muscle and bone mass, muscle strength, balance, and functional capacity [45]. As people age, the musculoskeletal system becomes less efficient at using dietary protein and its amino acids [8]. Therefore, expert consensus recommends at least 1.2–1.5 g/kg/day of protein for older adults, distributed throughout the day with 25–30 g per meal to further promote muscle protein synthesis [24]. Additionally, consuming 2.5–3 g of leucine per meal can stimulate muscle contractile protein building [46]. Supplementing with leucine (the precursor of TRP) or vitamin D can further enhance muscle synthesis and functional capacity [24]. Additional nutritional measures include a daily calcium intake of 1000–1300 mg, which should be provided through supplementation if dietary intake is insufficient [47]. Recognized as a key factor for bone and muscle health, vitamin D facilitates calcium and phosphate absorption and regulates calcium‐dependent processes in muscle, such as contractility, mitochondrial function, and insulin sensitivity [48]. To achieve a good target, it may be necessary to supplement it with at least 1000 IU/day [8]. Regarding this topic, a previous study showed that supplementing older sarcopenic adults with mobility limitations with vitamin D plus leucine‐enriched whey protein may reduce the progression of chronic low‐grade inflammation [49]. Furthermore, supplementation with 3 g/daily of calcium salt form of β‐hydroxy‐β‐methylbutyrate (Ca‐HMB) has demonstrated some benefits/efficacy in sarcopenia management, principally when combined with resistance exercise after a recent hospitalization [24, 25, 26, 27, 28, 29, 30, 31, 32, 33, 34, 35, 36, 37, 38, 39, 40, 41, 42, 43, 44, 45, 46, 47, 48, 49, 50].

Evidence supports the role of non‐pharmacological interventions in the management of osteosarcopenia. The FrOST study demonstrated that high‐intensity resistance training combined with protein supplementation for 18 months significantly improved skeletal muscle index, handgrip strength, gait velocity, and areal bone mineral density at the lumbar spine and total hip in older men with osteosarcopenia [51]. Similarly, Banitalebi et al. reported that 12 weeks of elastic‐band resistance training in women with osteosarcopenic obesity improved functional outcomes such as handgrip strength and the 30 s chair stand test, although no significant changes were observed in bone mineral density [52]. Consistent with these findings, a review of randomized controlled trials by Atlihan et al. found moderate‐quality evidence that progressive resistance training improves muscle mass, strength, and muscle quality in older adults with osteosarcopenia and may help maintain or increase bone mineral density [53].

It is important to note that currently, testosterone treatment is also not approved for sarcopenia in the absence of clear hypogonadism symptoms. For patients with these conditions, the use of testosterone has a beneficial impact on muscle mass and strength [44]. Other androgenic medications under investigation include Selective Androgen Receptor Modulators (SARMs) and myostatin inhibitors, which have tissue‐selective androgenic effects that can improve lean body mass by 3%–5% with minimal adverse effects. Additionally, research has highlighted some promising anti‐catabolic drugs that could reduce systemic inflammation, while Growth Hormone (GH) is also considered a potential treatment for sarcopenia, capable of increasing muscle mass, although it may not necessarily enhance muscle strength or function. Furthermore, various stem cell populations show potential for muscle repair and regeneration. Emerging therapies have demonstrated regenerative capacity and improvements in muscle function through direct differentiation and paracrine effects, including secretion of growth factors and anti‐inflammatory molecules [24].

Unexpected benefits in muscle function in older adults include improved physical performance and a slower decline in muscle strength, such as walking speed and lower extremity strength. These effects have been linked to Angiotensin‐Converting Enzyme Inhibitors, which seem to work by enhancing muscle blood flow and directly affecting muscle metabolism, and also to β2‐adrenergic receptor agonists, which have shown potential to increase muscle protein synthesis and reduce protein degradation [24]. Additionally, antidiabetic drugs (such as GLP‐1 receptor agonists, SGLT‐2 inhibitors, and metformin) have demonstrated potential for improving muscle mass, function, and insulin sensitivity, particularly in patients with metabolic disorders [24]. Finally, creatine has also shown potential to improve BMD in some studies, influence the activity of cells involved in both bone formation and resorption, and decrease the risk of falls in older adults. Preliminary evidence suggests that creatine may have anti‐inflammatory effects when elevated metabolic stress occurs, such as during intense aerobic exercise [42].

## 7 Conclusion

In summary, osteosarcopenia is a significant geriatric syndrome with considerable clinical and socioeconomic effects. Its pathophysiology underscores the strong link between bone and muscle through mechanical, metabolic, and endocrine pathways, resulting in worse outcomes than those seen with osteoporosis or sarcopenia alone. Despite increasing recognition of osteosarcopenia as a clinically relevant geriatric syndrome, it remains a construct in consolidation rather than a fully operationalized disease entity.

While advances in metabolic, molecular, and regenerative therapies are promising, robust evidence supporting their clinical application is still lacking. Standardized diagnostic criteria, longitudinal cohort studies, and well‐designed randomized controlled trials with combined bone and muscle endpoints are essential to translate emerging pathophysiological insights into effective clinical interventions. Meanwhile, comprehensive management should focus on dual‐action strategies, both pharmacological and nonpharmacological, combining adequate intake of protein, calcium, and vitamin D with resistance training and bone‐strengthening exercises. Incorporating muscle and bone assessments into routine fracture prevention and geriatric care can help reduce falls, fractures, morbidity, and healthcare costs, thereby improving outcomes in aging populations.

## Author Contributions

Erik Luiz Bonamigo, Marcella Nora Maia, Renata Pinheiro Cavallaro de Oliveira, Régis Gemerasca Mestriner, and Gustavo Duque drafted the first version of the manuscript; Erik Luiz Bonamigo provided the figures. Gustavo Duque conceptualized the article and revised the first draft. All authors contributed critical feedback, helped to structure the manuscript, and approved the final version.

## Funding

The authors have nothing to report.

## Ethics Statement

This manuscript is an original, comprehensive review of the literature and has not been published elsewhere. All sources have been properly cited and attributed.

## Conflicts of Interest

The authors declare no conflicts of interest.

## Data Availability

Data sharing not applicable to this article as no datasets were generated or analysed during the current study.

## References

[1] Y. Pu , Y. Teng , Y. Li , et al., “Osteosarcopenia: Key Molecular Mechanisms and Translational Perspectives,” Frontiers in Physiology 16 (2026): 1723522, 10.3389/fphys.2025.1723522. 41584796 PMC12827509

[2] Y. Long , H. Zhao , D. Sun , Y. Wen , and Y. Yu , “Association Between Osteosarcopenia and Frailty in Older Adults: A Systematic Review and Meta‐Analysis,” BMC Geriatrics 26, no. 1 (2026): 372, 10.1186/s12877-026-06976-z. 41580607 PMC13003619

[3] G. Fagundes Belchior , B. Kirk , E. A. Pereira da Silva , and G. Duque , “Osteosarcopenia: Beyond Age‐Related Muscle and Bone Loss,” European Geriatric Medicine 11, no. 5 (2020): 715–724, 10.1007/s41999-020-00355-6. 32676865

[4] J. Xu , E. M. Reijnierse , J. Pacifico , C. S. Wan , and A. B. Maier , “Sarcopenia Is Associated With 3‐Month and 1‐Year Mortality in Geriatric Rehabilitation Inpatients: RESORT,” Age and Ageing 50, no. 6 (2021): 2147–2156, 10.1093/ageing/afab134. 34260683 PMC8581377

[5] A. García‐Hermoso , I. Cavero‐Redondo , R. Ramírez‐Vélez , et al., “Muscular Strength as a Predictor of All‐Cause Mortality in an Apparently Healthy Population: A Systematic Review and Meta‐Analysis of Data From Approximately 2 Million Men and Women,” Archives of Physical Medicine and Rehabilitation 99, no. 10 (2018): 2100–2113.e5, 10.1016/j.apmr.2018.01.008. 29425700

[6] B. Kirk , P. M. Cawthon , H. Arai , et al., “The Conceptual Definition of Sarcopenia: Delphi Consensus From the Global Leadership Initiative in Sarcopenia (GLIS),” Age and Ageing 53, no. 3 (2024): afae052, 10.1093/ageing/afae052. 38520141 PMC10960072

[7] J. Pacifico , E. M. Reijnierse , W. K. Lim , and A. B. Maier , “The Association Between Sarcopenia as a Comorbid Disease and Incidence of Institutionalisation and Mortality in Geriatric Rehabilitation Inpatients: REStORing Health of Acutely Unwell adulTs (RESORT),” Gerontology 68, no. 5 (2022): 498–508, 10.1159/000517461. 34340238

[8] B. Kirk , J. Zanker , and G. Duque , “Osteosarcopenia: Epidemiology, Diagnosis, and Treatment—Facts and Numbers,” Journal of Cachexia, Sarcopenia and Muscle 11, no. 3 (2020): 609–618, 10.1002/jcsm.12567. 32202056 PMC7296259

[9] World Health Organization , World Report on Ageing and Health (WHO, 2015).

[10] Consensus Development Conference: Diagnosis, Prophylaxis, and Treatment of Osteoporosis,” American Journal of Medicine 94, no. 6 (1993): 646–650, 10.1016/0002-9343(93)90218-e. 8506892

[11] J. A. Kanis , L. J. Melton , C. Christiansen , C. C. Johnston , and N. Khaltaev , “The Diagnosis of Osteoporosis,” Journal of Bone and Mineral Research 9, no. 8 (2009): 1137–1141, 10.1002/jbmr.5650090802. 7976495

[12] M. Srivastava and C. Deal , “Osteoporosis in Elderly: Prevention and Treatment,” Clinics in Geriatric Medicine 18, no. 3 (2002): 529–555, 10.1016/s0749-0690(02)00022-8. 12424871

[13] A. M. Wu , C. Bisignano , S. L. James , et al., “Global, Regional, and National Burden of Bone Fractures in 204 Countries and Territories, 1990–2019: A Systematic Analysis From the Global Burden of Disease Study 2019,” Lancet Healthy Longevity 2, no. 9 (2021): e580–e592, 10.1016/S2666-7568(21)00172-0. 34723233 PMC8547262

[14] B. Gullberg , O. Johnell , and J. A. Kanis , “World‐Wide Projections for Hip Fracture,” Osteoporosis International 7, no. 5 (1997): 407–413, 10.1007/pl00004148. 9425497

[15] N. Binkley and B. Buehring , “Beyond FRAX: It's Time to Consider Sarco‐Osteopenia,” Journal of Clinical Densitometry 12, no. 4 (2009): 413–416, 10.1016/j.jocd.2009.06.004. 19733110

[16] H. He , Y. Liu , Q. Tian , C. J. Papasian , T. Hu , and H. W. Deng , “Relationship of Sarcopenia and Body Composition With Osteoporosis,” Osteoporosis International 27, no. 2 (2015): 473–482, 10.1007/s00198-015-3241-8. 26243357

[17] D. Scott , J. Johansson , L. B. McMillan , P. R. Ebeling , P. Nordstrom , and A. Nordstrom , “Associations of Sarcopenia and Its Components With Bone Structure and Incident Falls in Swedish Older Adults,” Calcified Tissue International 105, no. 1 (2019): 26–36, 10.1007/s00223-019-00540-1. 30899995

[18] World Health Organization , “Fragility Fractures,” (World Health Organization, 2024), https://www.who.int/news‐room/fact‐sheets/detail/fragility‐fractures.

[19] B. Kirk , S. Miller , J. Zanker , and G. Duque , “A Clinical Guide to the Pathophysiology, Diagnosis and Treatment of Osteosarcopenia,” Maturitas 140 (2020): 27–33, 10.1016/j.maturitas.2020.05.012. 32972632

[20] H. P. Hirschfeld , R. Kinsella , and G. Duque , “Osteosarcopenia: Where Bone, Muscle, and Fat Collide,” Osteoporosis International 28, no. 10 (2017): 2781–2790, 10.1007/s00198-017-4151-8. 28733716

[21] R. T. Lin , B. Osipov , D. Steffen , et al., “Saturated Fatty Acids Negatively Affect Musculoskeletal Tissues in Vitro and in Vivo,” Matrix Biology Plus 23 (2024): 100153, 10.1016/j.mbplus.2024.100153. 38882396 PMC11179588

[22] A. Al Saedi , S. Chow , S. Vogrin , G. J. Guillemin , and G. Duque , “Association Between Tryptophan Metabolites, Physical Performance, and Frailty in Older Persons,” International Journal of Tryptophan Research 15 (2022): 15, 10.1177/11786469211069951. PMC8808031 35125874

[23] J. Ballesteros , D. Rivas , and G. Duque , “The Role of the Kynurenine Pathway in the Pathophysiology of Frailty, Sarcopenia, and Osteoporosis,” Nutrients 15, no. 14 (2023): 3132, 10.3390/nu15143132. 37513550 PMC10383689

[24] X. Liu , X. Chen , and J. Cui , “Therapeutic Advances in Sarcopenia Management: From Traditional Interventions to Personalized Medicine,” Clinical Nutrition 11 (2025): 187–197, 10.1016/j.clnu.2025.06.007. 40580805

[25] Y. Dong , H. Yuan , G. Ma , and H. Cao , “Bone‐Muscle Crosstalk Under Physiological and Pathological Conditions,” Cellular and Molecular Life Sciences 81, no. 1 (2024): 310, 10.1007/s00018-024-05331-y. 39066929 PMC11335237

[26] O. Rosas‐Carrasco , B. Manrique‐Espinoza , J. C. López‐Alvarenga , B. Mena‐Montes , and I. Omaña‐Guzmán , “Osteosarcopenia Predicts Greater Risk of Functional Disability Than Sarcopenia: A Longitudinal Analysis of FraDySMex Cohort Study,” Journal of Nutrition Health & Aging 28, no. 11 (2024): 100368, 10.1016/j.jnha.2024.100368. PMC12879309 39307074

[27] L. A. Schaap , N. M. van Schoor , P. Lips , and M. Visser , “Associations of Sarcopenia Definitions, and Their Components, With the Incidence of Recurrent Falling and Fractures: The Longitudinal Aging Study Amsterdam,” Journals of Gerontology Series A 73, no. 9 (2017): 1199–1204, 10.1093/gerona/glx245. 29300839

[28] E. J. Metter , L. A. Talbot , M. Schrager , and R. A. Conwit , “Arm‐Cranking Muscle Power and Arm Isometric Muscle Strength Are Independent Predictors of All‐Cause Mortality in Men,” Journal of Applied Physiology 96, no. 2 (2004): 814–821, 10.1152/japplphysiol.00370.2003. 14555682

[29] A. Lee , C. McArthur , G. Ioannidis , et al., “Associations Between Osteosarcopenia and Falls, Fractures, and Frailty in Older Adults: Results From the Canadian Longitudinal Study on Aging (CLSA),” Journal of the American Medical Directors Association 25, no. 1 (2024): 167–176.e6, 10.1016/j.jamda.2023.09.027. 37925161

[30] N. Veronese , L. Smith , M. Barbagallo , et al., “Sarcopenia and Fall‐Related Injury Among Older Adults in Five Low‐ and Middle‐Income Countries,” Experimental Gerontology 147 (2021): 111262, 10.1016/j.exger.2021.111262. 33516908

[31] J. A. Kanis , C. Cooper , R. Rizzoli , and J. Y. Reginster , “European Guidance for the Diagnosis and Management of Osteoporosis in Postmenopausal Women,” Osteoporosis International 30, no. 1 (2019): 3–44, 10.1007/s00198-018-4704-5. 30324412 PMC7026233

[32] A. J. Cruz‐Jentoft , G. Bahat , J. Bauer , et al., “Sarcopenia: Revised European Consensus on Definition and Diagnosis,” Age and Ageing 48, no. 1 (2019): 16–31, 10.1093/ageing/afy169. 30312372 PMC6322506

[33] T. K. Malmstrom , D. K. Miller , E. M. Simonsick , L. Ferrucci , and J. E. Morley , “SARC‐F: A Symptom Score to Predict Persons With Sarcopenia at Risk for Poor Functional Outcomes,” Journal of Cachexia, Sarcopenia and Muscle 7, no. 1 (2015): 28–36, 10.1002/jcsm.12048. 27066316 PMC4799853

[34] S. N. Morin , W. D. Leslie , and J. T. Schousboe , “Osteoporosis,” JAMA 30 (2025): 894, 10.1001/jama.2025.6003. 40587168

[35] E. Mocini , C. Piciocchi , G. Defeudis , and S. Migliaccio , “Sarcopenia and Osteoporosis,” Gerontology 71, no. 8 (2025): 649–655, 10.1159/000546501. 40545813

[36] A. J. Mayhew , K. Amog , S. Phillips , et al., “The Prevalence of Sarcopenia in Community‐Dwelling Older Adults, an Exploration of Differences Between Studies and Within Definitions: A Systematic Review and Meta‐Analyses,” Age and Ageing 48, no. 1 (2018): 48–56, 10.1093/ageing/afy106. 30052707

[37] B. Vellas , R. A. Fielding , C. Bens , et al., “Implications of ICD‐10 for Sarcopenia Clinical Practice and Clinical Trials: Report by the International Conference on Frailty and Sarcopenia Research Task Force,” Journal of Frailty & Aging 7 (2017): 1–7, 10.14283/jfa.2017.30. 29412436

[38] G. Coletta and S. M. Phillips , “An Elusive Consensus Definition of Sarcopenia Impedes Research and Clinical Treatment: A Narrative Review,” Ageing Research Reviews 86 (2023): 101883, 10.1016/j.arr.2023.101883. 36792012

[39] N. Bonnet , L. Bourgoin , E. Biver , E. Douni , and S. Ferrari , “RANKL Inhibition Improves Muscle Strength and Insulin Sensitivity and Restores Bone Mass,” Journal of Clinical Investigation 129, no. 8 (2019): 3214–3223, 10.1172/jci125915. 31120440 PMC6668701

[40] C. Ceolin , C. Ziliotto , M. V. Papa , A. Bertocco , G. Sergi , and M. De Rui , “Beyond Bone Effects: The Role of Denosumab in Muscle Health ‐ A Systematic Review,” Aging Clinical and Experimental Research 38, no. 1 (2026): 46, 10.1007/s40520-025-03285-0. 41511732 PMC12819549

[41] D. C. Bauer and K. E. Ensrud , “Denosumab and Fracture Prevention in Primary Care Practice,” JAMA Internal Medicine 27 (2025): 876, 10.1001/jamainternmed.2025.1488. 40423959

[42] D. G. Candow , S. C. Forbes , P. D. Chilibeck , S. M. Cornish , J. Antonio , and R. B. Kreider , “Effectiveness of Creatine Supplementation on Aging Muscle and Bone: Focus on Falls Prevention and Inflammation,” Journal of Clinical Medicine 8, no. 4 (2019): 488, 10.3390/jcm8040488. 30978926 PMC6518405

[43] W. J. Chodzko‐Zajko , D. N. Proctor , M. A. Fiatarone Singh , et al., “Exercise and Physical Activity for Older Adults,” Medicine & Science in Sports & Exercise 41, no. 7 (2009): 1510–1530, 10.1249/mss.0b013e3181a0c95c. 19516148

[44] Y. Rolland , C. Dray , B. Vellas , and P. De Souto Barreto , “Current and Investigational Medications for the Treatment of Sarcopenia,” Metabolism 149 (2023): 155597, 10.1016/j.metabol.2023.155597. 37348598

[45] B. Kirk , K. Mooney , F. Amirabdollahian , and O. Khaiyat , “Exercise and Dietary‐Protein as a Countermeasure to Skeletal Muscle Weakness: Liverpool Hope University – Sarcopenia Aging Trial (LHU‐SAT),” Frontiers in Physiology 10 (2019): 445, 10.3389/fphys.2019.00445. 31133863 PMC6524700

[46] J. M. Bauer , S. Verlaan , I. Bautmans , et al., “Effects of a Vitamin D and Leucine‐Enriched Whey Protein Nutritional Supplement on Measures of Sarcopenia in Older Adults, the PROVIDE Study: A Randomized, Double‐Blind, Placebo‐Controlled Trial,” Journal of the American Medical Directors Association 16, no. 9 (2015): 740–747, 10.1016/j.jamda.2015.05.021. 26170041

[47] Institute of Medicine (US) Committee to Review Dietary Reference Intakes for Vitamin D and Calcium , Dietary Reference Intakes for Calcium and Vitamin D (National Academies Press (US), 2011). 21796828

[48] M. Fatima , S. L. Brennan‐Olsen , and G. Duque , “Therapeutic Approaches to Osteosarcopenia: Insights for the Clinician,” Therapeutic Advances in Musculoskeletal Disease 11 (2019): 1759720X19867009, 10.1177/1759720x19867009. PMC6686316 31431811

[49] K. Liberman , R. Njemini , Y. Luiking , et al., “Thirteen Weeks of Supplementation of Vitamin D and Leucine‐Enriched Whey Protein Nutritional Supplement Attenuates Chronic Low‐Grade Inflammation in Sarcopenic Older Adults: The PROVIDE Study,” Aging Clinical and Experimental Research 31, no. 6 (2019): 845–854, 10.1007/s40520-019-01208-4. 31049877 PMC6583678

[50] D. Meza‐Valderrama , D. Sánchez‐Rodríguez , M. Messaggi‐Sartor , et al., “Supplementation With β‐Hydroxy‐β‐Methylbutyrate After Resistance Training in Post‐Acute Care Patients With Sarcopenia: A Randomized, Double‐Blind Placebo‐Controlled Trial,” Archives of Gerontology and Geriatrics 119 (2023): 105323, 10.1016/j.archger.2023.105323. 38171034

[51] W. Kemmler , M. Kohl , F. Jakob , K. Engelke , and S. von Stengel , “Effects of High Intensity Dynamic Resistance Exercise and Whey Protein Supplements on Osteosarcopenia in Older Men With Low Bone and Muscle Mass. Final Results of the Randomized Controlled FrOST Study,” Nutrients 12, no. 8 (2020): 2341, 10.3390/nu12082341. 32764397 PMC7468852

[52] E. Banitalebi , M. Faramarzi , M. M. Ghahfarokhi , F. SavariNikoo , N. Soltani , and A. Bahramzadeh , “Osteosarcopenic Obesity Markers Following Elastic Band Resistance Training: A Randomized Controlled Trial,” Experimental Gerontology 135 (2020): 110884, 10.1016/j.exger.2020.110884. 32092502

[53] R. Atlihan , B. Kirk , and G. Duque , “Non‐Pharmacological Interventions in Osteosarcopenia: A Systematic Review,” Journal of Nutrition, Health & Aging 25, no. 1 (2020): 25–32, 10.1007/s12603-020-1537-7. PMC12879118 33367459

